# UNR/*CSDE1* Expression Is Critical to Maintain Invasive Phenotype of Colorectal Cancer through Regulation of c-MYC and Epithelial-to-Mesenchymal Transition

**DOI:** 10.3390/jcm8040560

**Published:** 2019-04-25

**Authors:** Javier Martinez-Useros, Nuria Garcia-Carbonero, Weiyao Li, Maria J. Fernandez-Aceñero, Ion Cristobal, Raul Rincon, Maria Rodriguez-Remirez, Aurea Borrero-Palacios, Jesus Garcia-Foncillas

**Affiliations:** 1 Translational Oncology Division, OncoHealth Institute, FIIS-Fundacion Jimenez Diaz University Hospital, Autonomous University of Madrid, 28040 Madrid, Spain; nuria.garciac@quironsalud.es (N.G.-C.); weiyao.li@quironsalud.es (W.L.); ion.cristobal@fjd.es (I.C.); rrincon23@gmail.com (R.R.); maria.rremirez@fjd.es (M.R.-R.); aureaborreropalacios@gmail.com (A.B.-P.); 2 Department of Pathology, Clinico San Carlos University Hospital, 28040 Madrid, Spain; mgg10167@gmail.com

**Keywords:** UNR, *CSDE1*, colorectal cancer, metastasis, prognosis biomarker, c-MYC

## Abstract

*CSDE1* (cold shock domain containing E1) gene is located upstream of the *N-RAS* locus, and codes for an RNA-binding protein named Upstream of N-Ras (UNR). In cancer, *CSDE1* has been shown to regulate c-Fos, c-Myc, Pten, Rac1, or Vimentin. UNR/*CSDE1* has been studied in breast, melanoma, pancreatic and prostate cancer. Then, the aim of this study is to evaluate the role of *CSDE1*/UNR in colorectal cancer progression and maintenance of aggressive phenotype. We firstly evaluated UNR/*CSDE1* expression in human colon cancer derived cell lines and patient samples. Subsequently, we performed functional experiments by UNR/*CSDE1* downregulation. We also evaluated UNR/*CSDE1* prognostic relevance in two independent sets of patients. Not only was UNR/*CSDE1* expression higher in tumor samples compared to untransformed samples, but also in colonospheres and metastatic origin cell lines than their parental and primary cell lines, respectively. Downregulation of UNR/*CSDE1* reduced cell viability and migration throughout a restrain of epithelial-to-mesenchymal transition and increases sensitivity to apoptosis. Interestingly, high UNR/*CSDE1* expression was associated with poor prognosis and correlated positively with c-MYC expression in colorectal cancer samples and cell lines. Here, we show for the first time compelling data reporting the oncogenic role of UNR/*CSDE1* in human colorectal cancer.

## 1. Introduction

Colorectal cancer (CRC) is one of the most common gastrointestinal malignant tumors in the world and it has one of the highest rates of morbidity and mortality worldwide, with 78,500 new cases of CRC expected to be diagnosed in men and 67,100 in women in 2019 [1].

Moreover, it is the third leading cause of cancer death in both sexes, with 27,640 estimated deaths in male and 23,380 in women in the USA in 2019 [1]. CRC has higher incidence in industrialized countries and this incidence is lower in patients under 50 years, but it increases considerably with age. On the other hand, in developed countries, like the USA, incidence has begun to decrease due the use of early detection methods in clinical practice, such as sigmoidoscopy and colonoscopy with polypectomy [2].

According to the model of CRC carcinogenesis proposed by Fearon and Volgestein, the loss of genomic stability can drive the development of CRC and facilitates the acquisition of multiple tumor-associated aberrations [3]. Nevertheless, this model shows that carcinogenesis requires additional molecular alterations [4]. In this regard, RNA binding proteins (RBPs) play a key role since they are pivotal components in the determination of mRNA and miRNA via the regulation of mRNA splicing, translation, or stability [5]. Alteration in expression or activity of RBPs has been reported in several malignancies [6].

In the late 1980s, an active transcription unit called UNR (upstream of *N-RAS*) was described in the 5′ flanking region of the *N-RAS* gene [7]. Indeed, UNR down-modulates N-ras through mRNA accumulation in different tissues [8], although UNR expression is not affected by the activation of N-ras [9]. In vitro assays indicated that UNR could interact with cytoplasmic RNA in a sequence-specific manner. This fact was due to the presence of five cold shock domains in its protein structure which gave the name to the UNR gene sequence (*CSDE1*, cold shock domain containing E1) [10]. These domains allow UNR to bind internal ribosome entry segments (IRES) of mRNA acting as an RNA chaperone to change their structure into a more functionally competent for translation [11]. The focus of this interoperability has pointed how UNR is able to regulate expression of different oncogenes like *c-FOS* [12,13], *c-MYC* [11,14], *VIM*, or *RAC1* [15]. To date, the role of UNR/*CSDE1* has been studied in melanoma [15], breast [16], prostate [17], and pancreatic cancer [18]. Furthermore, the *CSDE1* gene itself could be a target of proviral insertions of B-lymphomagenic virus Akv1-99, which led to an aberrant expression of the downstream *N-RAS* gene in B-cell lymphoma [9]. Despite the oncogenic functions of UNR/*CSDE1*, it plays a crucial role in cell development and homeostasis since homozygous knockout mice of *CSDE1* have not been obtained due the embryonic lethal effect of the deletion [8].

The purpose of this study is to evaluate, for the first time, the role of UNR/*CSDE1* in colorectal cancer carcinogenesis. A secondary aim of the study is to provide the clinical relevance of UNR/*CSDE1* expression as a potential biomarker for CRC progression.

## 2. Experimental Section

### 2.1. Human Cell Lines

Ten human-derived CRC cell lines obtained from the American Type Culture Collection (ATCC number): DLD1 (CCL-221), SW620 (CCL-227), SW480 (CCL-228), RKO (CRL-2577), LoVo (CCL-229), WiDr (CCL-218), LS513 (CRL-2134), HCT15 (CCL-225), HCT116 (CCL-247), and HT29 (HTB-38) were cultured with RPMI supplemented with 10% FBS, penicillin (100 U/mL)/streptomycin (100 U/mL) at 37 °C with a 5% CO_2_ atmosphere. Colonosphere-derived cell lines from DLD1 and LS513 where isolated as described by Bitarte et al. [19].

### 2.2. Patient Samples

A total of 74 human CRC patients who underwent surgery from 2007 to 2013 at the General and Digestive Tract Surgery Department from Fundacion Jimenez Diaz University Hospital were assessed for eligibility. Fresh-frozen samples from tumor and their paired adjacent untransformed tissue were obtained from 36 CRC patients and were used for total protein evaluation and mRNA expression; formalin-fixed paraffin embedded (FFPE) tissues from 35 CRC patients were used for UNR protein evaluation by immunohistochemistry (IHC) and association with patient survival; and three different tumor samples from 3 different patients were taken immediately after surgical resection to carry out ex vivo experiments. In addition, two fresh-frozen untransformed samples from healthy mucosa were used as controls in protein evaluation by Western blot. To evaluate protein expression by IHC a tissue microarray (TMA) was constructed with 35 FFPE patient samples using the MTA-1 tissue arrayer (Beecher Instruments; Tartu, Estonia). Each core (diameter, 1 mm) was punched from pre-selected FFPE tumor regions. These tissue cores were then inserted in a recipient paraffin block.

The clinical features of the CRC patients included in the study are summarized in Table 1. Our cohort was mainly male patients (52%) with a median age of 64 years (range 37–83 years). The main localization of tumors was in sigma (32%) followed by rectum (23%) and right colon (17%). Pathologic diagnosis revealed 83% of tumors were pT3N0 or pT3N1 and synchronous metastatic disease was found in 66% of cases. Differentiation grade was moderate in 46% and well-differentiated in 26% of tumors. Molecular analysis showed that 82% of tumors were mismatch repair-proficient and *RAS* wild-type in 69% of tumors.

### 2.3. Ethics Statement

All human samples were kindly supplied by the BioBank of the Fundacion Jimenez Diaz—Universidad Autonoma de Madrid (PT13/0010/0012). All patients gave written informed consent for the use of their biological samples for research purposes. The institutional review board (IRB) of the Fundacion Jimenez Diaz Hospital evaluated the study, granting approval on 9 December 2014 with approval number 17/14. Moreover, fundamental ethical principles promoted by Spain (LOPD 15/1999) and the European Union Fundamental Rights of the EU (2000/C364/01) were followed. In addition, all patient´s data were processed according to Declaration of Helsinki (last revision 2013) and Spanish National Biomedical Research Law (14/2007, of 3 July).

### 2.4. Western Blot

Total protein from CRC cell lines and normal human mucosa tissues was extracted with RIPA buffer supplemented with protease inhibitor cocktail (Roche; Basel, Switzerland). Samples were fractionated by SDS–polyacrylamide gel electrophoresis, transferred to nitrocellulose membranes (BioRad; Hercules, CA, USA), and proteins were detected using specific antibodies for UNR (ab96124 Abcam; Cambridge, UK), Actin (a1978, Sigma-Aldrich; St. Louis, MO, USA), and c-Myc (ab32, Abcam; Cambridge, UK). Horseradish peroxidase-linked sheep anti-mouse (NA931V) antibodies (GE-Healthcare; Chicago, IL, USA) were used as the secondary antibodies. Blots were developed with the Amersham ECL Prime Western Blotting Detection Reagent (GE-Healthcare; Chicago, IL, USA). Quantification of protein band densitometry was carried out using ImageJ 1.52a software (Wayne Rasband, NIH; Bethesda, MD, USA).

### 2.5. Quantitative Real-Time PCR

Total RNA was isolated from the cell lines and patient samples with TRIzol Reagent (Invitrogen; Waltham, MA, USA). cDNA was generated with the SuperScript-III reverse transcriptase enzyme (Invitrogen; Waltham, MA, USA) following the manufacturer’s instructions. Quantitative real-time PCR was performed in triplicate with TaqMan Universal PCR Master Mix (Thermo Fisher Scientific; Waltham, MA, USA) in the FAST 7500 Real Time PCR System (Thermo Fisher Scientific; Waltham, MA, USA). *CSDE1* cDNA was amplified with the TaqMan Assay Hs00918650_m1 and the results were normalized to GAPDH (TaqMan Assay Hs02758991_g1) (Thermo Fisher Scientific; Waltham, MA, USA). All experiments were performed in triplicate.

### 2.6. RNA Interference

Two independent short interfering RNAs (siRNA) against csde1 mRNA were used (Silencer Select pre-designed siRNA s15373 and s15374 (Thermo Fisher Scientific; Waltham, MA, USA). UNR/*CSDE1* downregulation was performed with 3.5 million cells from three different CRC cell lines, DLD1, SW620 and RKO, by transfecting 600 pmol of each siRNA or the Silencer Negative Control-1 siRNA (Thermo Fisher Scientific; Waltham, MA, USA) using Lipofectamine 2000 reagent (Invitrogen; Waltham, MA, USA). UNR protein expression was evaluated by Western blot the next 24 h and 48 h and by immunohistochemistry 24 h after downregulation.

### 2.7. Cell Viability and Apoptosis

Cell viability after UNR/*CSDE1* downregulation was determined with a colorimetric reaction the 3-(4,5-dimethyl-thiazol-2yl)-5-(3-carboxymethoxyphenyl)-2-(4-sulfophenyl)-2H-tetrazolium (MTS) reduction assay following the manufacturer’s instructions (Promega; Madison, WI, USA). Apoptosis was assessed with the Annexin-V-FITC Apoptosis Detection Kit (BD Biosciences; San Jose, CA, USA) according to the manufacturer’s protocol after UNR/*CSDE1* downregulation alone or in combination of camptothecin for (SN38, IC50 = 50 nM) 24 h [20]. Cell cytometry was performed on a FACS Canto II (BD Biosciences; San Jose, CA, USA) and analyzed with FACS Diva software (BD Biosciences; San Jose, CA, USA). Three independent experiments were done and all experiments were performed in triplicate wells.

### 2.8. Invasion Assay

Invasion ability of tumor cells was estimated independently by wound healing and Boyden chamber assays. For wound healing assay cells lines were grown as a monolayer to complete or near confluence, and an artificial homogenous wound was done with a sterile plastic 10 μL micropipette tip. Images were captured at 0, 6, 12, 24, and 48 h during the experiment. The images were compared to quantify the invasion rate of the cells. Boyden chamber assay was performed in cell culture inserts with 8-µm pores in 24-well plates (Transwell, Costar, Corning; New York, USA) [21]. The migration index was determined as the migrated cells ratio relative to siRNA control transfected cells. Three independent experiments were done and all experiments were performed in triplicate.

### 2.9. Ex Vivo Assay

Three tumor samples from three different patients were taken immediately after surgical resection. Each sample was divided in two pieces, transferred onto a 12-well plate and cultured in DMEM (Dulbecco’s Modified Eagle Medium) (Gibco; Waltham, MA, USA) supplemented with 10% FBS (Fetal Bovine Serum), Penicillin (100 U/mL)/Streptomycin (100 U/mL). One of the tumor pieces was treated with camptothecin (SN38, 50 nM) (Sigma-Aldrich; St. Louis, MO, USA), whereas the other half remained untreated. After 24 h, the tissues were FFPE for IHC staining.

### 2.10. Immunohistochemistry

The procedure was conducted in 3 µm sections of tissues, TMA or FFPE cell lines. First, slides were deparaffinized by incubation at 60 °C for 10 min and antigen retrieval was performed on PT-Link (Dako; Glostrup, Denmark) for 20 min at 95 °C in a high pH buffered solution. To block endogenous peroxidase, holders were incubated with peroxidase blocking reagent (Dako; Glostrup, Denmark). Samples were then incubated for 20 min with a 1:50 dilution of UNR/*CDSE1* antibody (ab96124, Abcam; Cambridge, UK), 1:100 of cleaved Caspase-3 (9664, Cell Signaling; Danvers, MA, USA), 1:150 of Ki67 (clone SP6, Master Diagnostica; Granada, Spain), 1:1 of Vimentin (Clone V9, Dako Omnis; Glostrup, Denmark) and 1:1 of Cytokeratin 20 (GA777, Dako Omnis; Glostrup, Denmark), 1:500 β-Catenin (610153, BD Biosciences; San Jose, CA, USA), 1:50 Pan TGF-β (AB-100-NA, R&D Systems; Minneapolis, MN, USA), 1:50 Snail (3879S, Cell Signaling; Danvers, MA, USA) and 1:50 Slug (9585S, Cell Signaling; Danvers, MA, USA) followed by incubation with the appropriate anti-Ig horseradish peroxidase-conjugated polymer (EnVision, Dako; Glostrup, Denmark) to detect antigen-antibody reaction. All primary antibodies and anti-Ig horseradish peroxidase-conjugated secondary antibody presented high specificity and no positiveness resulted from these antibodies individually. A human intestinal tissue was used as a positive control for UNR immunohistochemical staining (according to the Human Protein Atlas available at http://www.proteinatlas.org). Sections were then visualized with 3,3′-diaminobenzidine (DAB) as a chromogen and counterstained with hematoxylin. Photographs were taken with a stereo microscope (Leica; Wetzlar, Germany).

To evaluate the expression of all antigens, DAB intensity was quantified with Image J (Fiji) software according to Fuhrich et al. [22].

To quantify the UNR immunostaining, a semiquantitative HistoScore (Hscore) was calculated. The Hscore was determined by estimation of the percentage of cells positively stained with low, medium, or high intensity of staining, after applying a weighting factor to each estimate. The following formula was used: Hscore = (low %) × 1 + (medium %) × 2 + (high %) × 3 and the results ranged from 0–300. Quantification for each patient biopsy was calculated with the average of both cores by two independent researchers (J.M.-U. and M.J.F.-A.)

### 2.11. Statistical Analysis

Nonparametric tests, Mann–Whitney U tests, or Kruskal–Wallis tests, were used to compare differences between two or more independent groups with small set of observations (*n* < 10) that are considered not normally distributed. To assess statistical analysis between UNR and c-MYC protein expression a ratio UNR vs. actin, and c-MYC vs. actin was assessed. Then, a Kolmogorov–Smirnov test confirmed that previous ratios were well-modelled by a normal distribution. Linear correlation between UNR and c-MYC ratios was evaluated and interpreted by Pearson’s r according to Cohen et al. [23]. Overall survival of CRC patients was defined as the interval between the dates of diagnosis and death from any cause. ROC (receiver operating characteristic) analysis was performed to identify the optimal cut-off point of UNR Hscore according to overall survival to separate into low- and high-risk CRC patients. The elected cut-off point presented the higher area under the curve (AUC) and an optimum sensitivity and specificity (Supplementary Figure S1). Survival curves were estimated using the Kaplan–Meier method and significant survival differences between groups were determined by the log-rank test. A *p*-value ≤ 0.05 indicated statistical significance. All statistics were performed with the IBM SPSS statistics 20.0 (Armonk, NY, USA).

### 2.12. TCGA-Colorectal Cancer Dataset Analysis

A TCGA dataset with 640 CRC patients was obtained from cBioPortal [24,25] and was used as an independent set of patients (cohort II). To assess survival analysis, only patients with available data of *CSDE1* expression and overall survival (*n* = 222) or progression-free survival (*n* = 193) were included. For both progression-free and overall survival, ROC curves did not show a clear cut-off point (AUC = 0.552, *p* = 0.382; and AUC = 0.555, *p* = 0.278, respectively). Therefore, to identify low- and high-risk patients according to the *CSDE1* Z-Score, the best cut-off point was determined by the 85th percentile of Z-score expression, considering the remaining 15% of patients with high *CSDE1* expression levels.

## 3. Results

### 3.1. UNR/CSDE1 Is Overexpressed in CRC But Not in Untransformed Tissues

**Figure 1:**
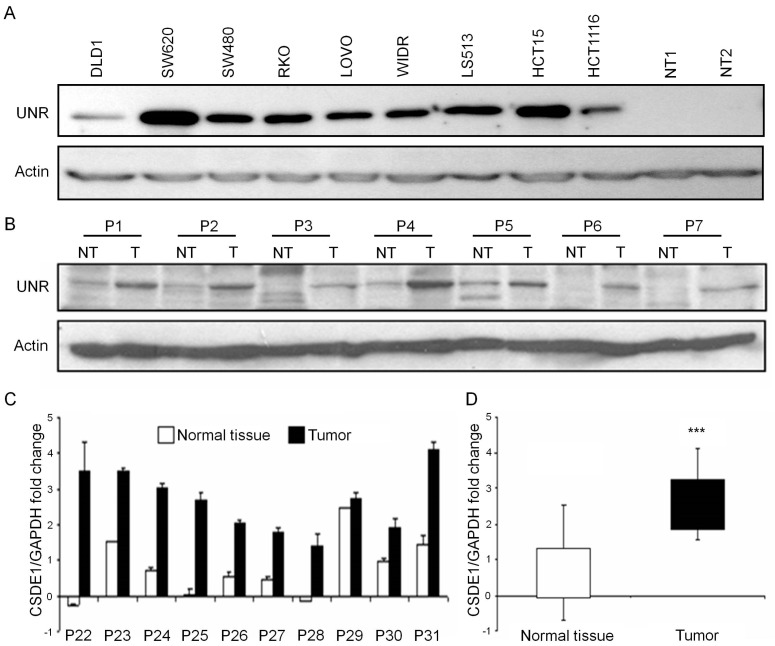
UNR/*CSDE1* is overexpressed in CRC cell lines and tumor samples from patients. (**A**) UNR was overexpressed in all human derived colorectal cancer cell lines compared with two human untransformed mucosa tissues (NT1, NT2). (**B**) Seven representative pair of protein samples from 21 independent paired of patient tissues (P1–P7) were analyzed by Western blot, showing UNR overexpression in tumor samples (T) compared to their adjacent normal tissues (NT). (**C**) Ten independent paired of samples, tumor and untransformed tissues (NT) from patients diagnosed with CRC were analyzed by Real Time PCR. *CSDE1* mRNA was overexpressed in most of tumor samples. (**D**) Samples were grouped by origin and statistical analysis was assessed (*** *p* < 0.001).

UNR protein levels were analyzed in a panel of nine human-derived CRC cell lines and compared with the expression in two untransformed mucosa tissues. All CRC-derived cell lines showed expression of UNR compared to mucosa tissues that showed a lack of expression. Indeed, almost all CRC-derived cell lines showed UNR overexpression, while two cell lines established from primary tumor presented the lowest expression (DLD1 and HCT116) (Figure 1A).

To verify UNR expression in human CRC samples, 21 tumor samples and their paired untransformed adjacent tissues before initiation of treatment were selected for protein evaluation. The 48% of tumors (*n* = 10/21) showed higher UNR expression than their untransformed adjacent tissues, while only 4% of tumors (*n* = 1/21) presented lower expression levels. The 48% of tumors (*n* = 10/21) showed no changes in UNR protein expression (Figure 1B). To verify this result, 20 independent samples (10 tumor samples and 10 paired untransformed samples) were analyzed by real-time PCR. Interestingly, we found *CSDE1* mRNA upregulation in 9/10 tumor samples compared to their untransformed samples (Figure 1C). Samples were then grouped according to their tumor and untransformed origin, and statistical analysis revealed a significant upregulation of *CSDE1* mRNA in tumor samples compared to their untransformed samples (*p* = 0.0004) (Figure 1D). These results support that high expression of UNR/*CSDE1* is a common event associated to tumor cells.

### 3.2. UNR/CSDE1 Knockdown Decreases Invasive Phenotype of CRC by Regulation of Epithelial-to-Mesenchymal Transition

**Figure 2:**
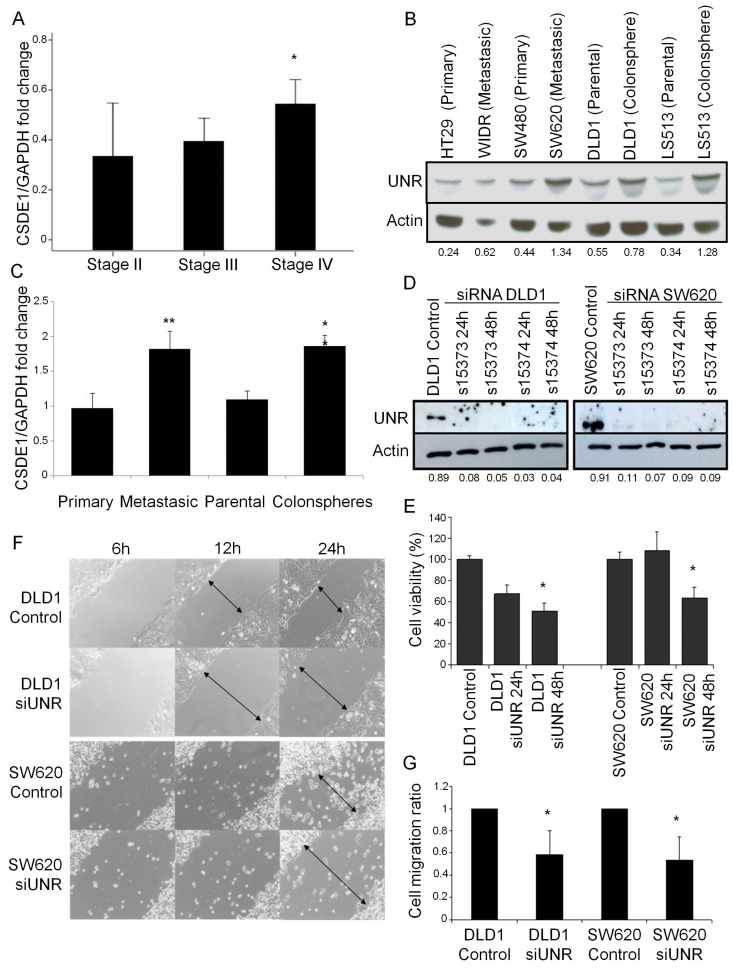
UNR/*CSDE1* promotes metastatic phenotype in CRC. (**A**) *CSDE1* mRNA expression of eight stage II tumor samples, 16 stage III tumor samples, and six stage IV tumor samples by qRT-PCR. (**B**) UNR protein expression of primary and metastatic CRC cell lines and parental and colospheres derived CRC cell lines. (**C**) *CSDE1* mRNA expression of CRC derived cell lines from (**B**) grouped by origin. (**D**) Two different siRNAs of csde1 (si15373 and si15374) were used to downregulate UNR protein expression in DLD1 and SW620 CRC derived cell lines. We verified UNR expression levels by western blot at 24 and 48 h after transfection. (**E**) MTS assay showed that both DLD1 and SW620 cell lines decreased cell viability at 48 h after UNR downregulation. (**F**) Microscope images of wound healing assay showed a reduced cell migration after UNR downregulation in both cell lines. Representative images taken at 6, 12, and 24 h after scratching. Arrows represent distance between cell migration heads. (**G**) Cell invasion assays performed in Boyden chamber showed that UNR silencing decreased invasion ability of both cell lines. UNR/Actin ratio is represented under Actin protein bands. All assays were performed with two different siRNA sequences. (* *p* < 0.05; ** *p* < 0.01).

Noting previous results, we decided to study whether UNR/*CSDE1* expression could be associated with initial stages or advanced tumors. To this aim, we quantified *CSDE1* mRNA expression levels in stage II (*n* = 16), stage III (*n* = 11), and metastatic stage IV tumors (*n* = 7). Here, we observed that metastatic tumor samples presented higher expression levels of *CSDE1* than stages II or III. Interestingly, *CSDE1* expression associated significantly to metastatic disease in CRC (*p* = 0.039) (Figure 2A).

Since colonspheres are an appropriate 3D in vitro cancer model and have formerly demonstrated their association with tumor cell undifferentiated phenotype and aggressiveness [26], we decided to evaluate UNR/*CSDE1* expression in different origin CRC derived cell lines. For this, CRC cell lines were analyzed for both protein and mRNA expression levels: two from primary origin (HT29 and SW480), two from metastatic origin (WiDr and SW620) and two colonspheres derived cell lines (DLD1 and LS513) with their parental cell lines (Figure 2B,C). UNR protein expression was not only higher in those cell lines from metastatic origin (WiDR and SW620) than those from primary origin, but also was higher in colonspheres derived cell lines (DLD1 and LS513) compared to their parental cell lines (Figure 2B). Subsequently, we evaluate *CSDE1* mRNA expression of these CRC cell lines. Expression data from individual cell line were grouped according to their different origin and statistical analysis was assessed. Interestingly, we found statistical significant differences in *CSDE1* expression between primary and metastatic cell lines (*p* = 0.004), and between parental and colonspheres (*p* = 0.004) (Figure 2C). Therefore, this result supports the fact that higher expression of UNR/*CSDE1* is associated with a metastatic and undifferentiated phenotype.

To further study whether UNR/*CSDE1* could be involved in cell survival and migration abilities we performed functional experiments with two different CRC cell lines, DLD1 and SW620. UNR expression was efficiently downregulated from both cell lines using two different small interfering RNAs for 24 and 48 h after transfection (Figure 2D). Cell viability was decreased significantly from 24 to 48 h after downregulation in DLD1 (*p* = 0.029), and after 48 h in SW620 (*p* = 0.042) that suggest the potential role of UNR to maintain tumor cell survival (Figure 2E). Wound healing assay revealed a delay in both cell lines from 12 h after silencing in DLD1 and after 24 h in SW620 compared to controls (Figure 2F). Migration assay performed by Boyden chamber assay confirmed these results and significant reductions in the migration ability of DLD1 (*p* = 0.049) and SW620 (*p* = 0.023) were observed (Figure 2G).

**Figure 3:**
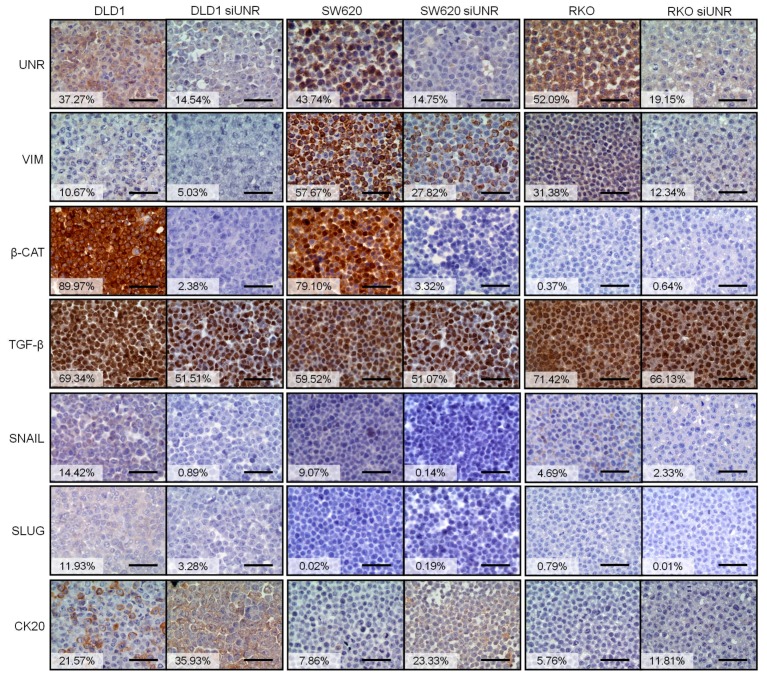
UNR is critical for the maintenance of epithelial-to-mesenchymal transition. Immunohistochemical staining for the expression of UNR, vimentin (VIM), β-catenin (β-CAT), TGF-β, snail, slug, and cytokeratin 20 (CK20) in DLD1, SW620, and RKO CRC derived cell lines before and after UNR/*CSDE1* downregulation. Vimentin (VIM), β-catenin (β-CAT), TGF-β, snail, and slug decreased their expression after UNR downregulation, while Cytokeratin 20 (CK20) expression increased after UNR downregulation. Cells were stained 48 h after UNR downregulation. Percentages of DAB staining for each CRC derived cell line are reflected in the white boxes. Scale bars represent 500 µm.

To study how UNR expression affects invasive capabilities of tumor cells, we evaluated the expression of different markers involved in epithelial-to-mesenchymal transition (EMT) in three different downregulated CRC-derived cell lines (DLD1, SW620, and RKO). Interestingly, protein expression of mesenchymal markers vimentin, β-catenin, and TGF-β reduced their expression after UNR downregulation. Only RKO did not express β-catenin, and no changes were observed after UNR downregulation. We also observed a reduced expression of EMT transcription factors snail and slug. However, expression of epithelial marker cytokeratin 20 increased after UNR downregulation in all tested CRC derived cell lines (Figure 3). These results highlight the crucial role that UNR/*CSDE1* plays in the migration ability of CRC tumor cells through regulation of EMT.

### 3.3. UNR/CSDE1 Downregulation Increases Response to Apoptosis Mediated by Camptothecin

**Figure 4:**
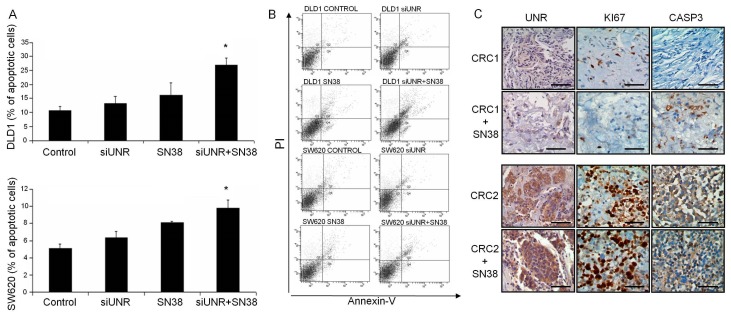
UNR influence camptothecin response in CRC derived cell lines and patients. (**A**) Bar diagram shows the percentage of both early and late apoptotic cells stained positive for Annexin-V after different treatments. DLD1 and SW620 cell lines were UNR downregulated and/or treated with camptothecin (SN38). Combination of UNR downregulation and camptothecin treatment increased apoptosis compared to individual treatments in DLD1 (up) or SW620 (down). (**B**) Representative dot blots from flow cytometry acquisition after propidium iodide (PI) and Annexin-V stained DLD1 and SW620 cells lines treated as previously. (**C**) Immunohistochemical staining for UNR, Ki67, and cleaved caspase 3 expression in two representative ex vivo assays from colorectal cancer (CRC) fresh tumor samples with the lowest UNR expression (CRC1) and the highest (CRC2) UNR expression levels. Camptothecin treatment did not affect UNR expression, but a reduction in Ki67 and a substantial increase in cleaved caspase 3 expression were observed in CRC1 compared with CRC2. No changes on Ki67 or cleaved caspase 3 were evidenced in CRC2. (* *p* ≤ 0.05). Scale bars represent 100 µm.

On the other hand, we wondered whether UNR/*CSDE1* could be involved in response to apoptosis stimuli in CRC to identify its translational potential use. To evaluate apoptosis induction CRC derived cell lines were treated with camptothecin according to Wang et al. [27]. Then, logarithmically-growing DLD1 and SW620 cell lines were treated with IC50 dose of camptothecin (SN38, 50 nM). Statistical analysis revealed significant differences between groups of treatment not only in DLD1 (*p* = 0.017), but also in SW620 (*p* = 0.043) (Figure 4A,B). UNR downregulation *per se* increased significantly apoptosis of DLD1 cell line (*p* = 0.050), but it was not enough to produce significant apoptotic stimulus in SW620 cell line (*p* = 0.825). Nevertheless, when UNR downregulation was combined with camptothecin treatment apoptosis increased significantly compared to control or individual treatments in DLD1 cell line (*p* = 0.050). However, combination of treatments in SW620 only achieved statistical significance compared to the untreated control (*p* = 0.046) or compared to UNR downregulation (*p* = 0.050), but not when treated with camptothecin treatment (*p* = 0.184) (Figure 4A,B).

The link between UNR expression and apoptosis induction through camptothecin treatment was confirmed with three independent tumor samples cultured ex vivo. Fresh tumor samples were treated with camptothecin and stained for UNR, Ki67, and cleaved caspase-3 protein detection. UNR expression levels were similar between untreated and treated samples indicating that camptothecin did not affect its expression. A tumor sample showed a substantial reduction in Ki67 and an increase in cleaved caspase-3 expression after treatment (CRC1) (Figure 4C). Remarkably, this sample had the lowest UNR expression levels. The other two tumor samples showed higher UNR expression levels and we did not find differences in any of three analyzed markers (CRC2) (Figure 4C). These results suggest the potential use of UNR/*CSDE1* downregulation in combination with chemotherapy to increase apoptosis of CRC tumor cells.

### 3.4. High UNR/CSDE1 Expression Levels Predict Poor Outcome of CRC Patients

**Figure 5:**
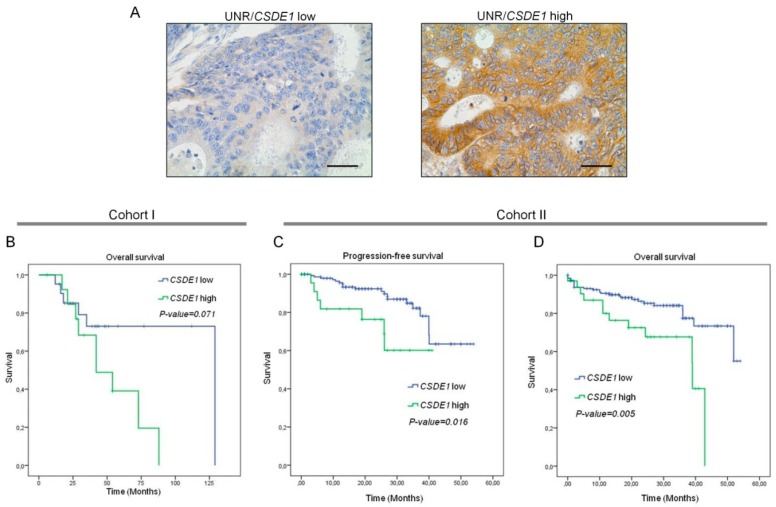
UNR/*CSDE1* expression predicts poor prognosis in CRC patients. (**A**) Representative micrographs of a low- (**left**) and high- (**right**) UNR expression tumors. Scale bars: 100 µm. (**B**) Kaplan-Meier curve for overall survival analysis of CRC patients that conforms cohort I. (**C**,**D**) Kaplan-Meier curves of cohort II for progression-free and overall survival analysis, respectively. *p*-value has been obtained by log-rank test.

We constructed and stained a TMA to detect UNR protein expression to determine its association with survival. All samples that stained positive for UNR exhibited a cytoplasmic expression pattern and membrane localization especially in some cases with high expression levels (Figure 5A right). Then, we stratified tumor samples into two groups, low- or high-UNR expression according to Hscore with the maximum AUC (0.45) (Supplementary Figure S1A) given by the ROC curve (Supplementary Figure S1B). Although no statistically significant association was found between UNR expression and overall survival of CRC patients, a high trend toward significance was observed (*p* = 0.071) (Figure 5B). Indeed, patients with high UNR expression presented shorter mean survival (52 months; 95% CI: 37–68 months) than patients with low expression (100 months; 95% CI: 76–124 months). Since the sample size of cohort I was too small (*n* = 35), we sought for a dataset of patients wide enough to validate the prognostic significance of high UNR expression. Therefore, an independent cohort was taken from The Cancer Genome Atlas (TCGA). Progression-free survival of patients with available *CSDE1* expression data (*n* = 193) demonstrated that high *CSDE1* expression levels presented shorter mean progression-free survival (30 months; 95% CI: 24–37 months) than patients with low *CSDE1* expression (45 months; 95% CI: 42–49 months) (*p* = 0.016) (Figure 5C). Indeed, overall survival analysis with 222 patients supported previous results. Here, those patients with high *CSDE1* expression levels showed shorter mean overall survival (31 months; 95% CI: 25–37 months) compared to those with low high UNR expression levels (45 months; 95% CI: 42–48 months) (*p* = 0.005) (Figure 5D). Altogether, the results obtained from cohort II support the malignant role of UNR/*CDSE1* expression in CRC.

### 3.5. UNR/CSDE1 Expression Correlated with c-MYC Expression in CRC

**Figure 6:**
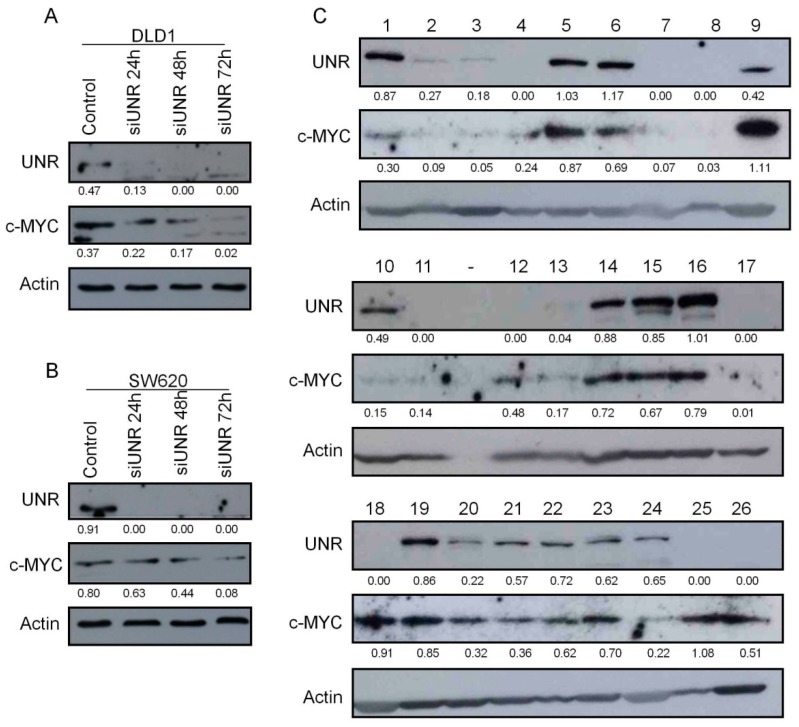
c-MYC protein expression is associated to UNR protein expression in CRC derived cell lines and patients. (**A**,**B**) UNR and c-MYC protein expression in DLD1 and SW620 cell lines were evaluated at 24, 48, and 72 h after UNR downregulation. (**C**) Evaluation of UNR and c-MYC protein expression in 26 human CRC tumor samples (1–26). Protein bands’ quantification is shown under each band.

Since UNR protein has the ability to bind c-myc RNA, which contributes to stimulate c-myc translation [11,14], we wonder whether c-MYC protein expression was linked to UNR expression in CRC. To test this hypothesis in vitro, UNR was downregulated from DLD1 and SW620 cells lines, then c-MYC expression was evaluated. Interestingly, we found a decrease in c-MYC expression gradually from 24 h in both CRC cell lines, and reached its maximum downregulation within 72 h after UNR knockdown (Figure 6A,B). To verify whether this connection occurs in vivo, UNR and c-MYC protein expression were evaluated in 26 fresh-frozen CRC patient samples (Figure 6C). Statistical analysis performed with both protein/Actin ratios exhibited a moderate significant positive correlation (Pearson´s *r* = 0.432; *p* = 0.028). These results support the notion that UNR/*CSDE1* may promote the malignant phenotype through regulation of c-MYC proto-oncogen in CRC.

## 4. Discussion

Post-transcriptional gene regulation is a rapid and efficient way to adjust the proteome of a cell to environments in constant variation. Most RBPs, together with their targets, form complex networks that play crucial role in cell proliferation, differentiation, invasion, metastasis, and apoptosis [5]. Some of these RBPs which have been largely studied in cancer development are Sam68, eIF4E, or HuR. Sam68 promotes inclusion of exon v5 in the CD44 pre-mRNA, which encodes a cell surface protein involved in cancer [28]. Sam68 is overexpressed in prostate, breast, renal, and cervical human cancer [29,30]. EIF4E binds to the m7GTP-cap structure present at the 5′ end of mRNAs and is essential for mRNA translation [31]. It is also overexpressed in different tumor types, like colon, prostate, breast, lung, or gastric cancer [32], and eIF4E is often associated to poor prognosis and malignancy [33,34]. Perhaps the most studied RBP involved in cancer is human antigen R (HuR), a protein that regulates the translation and stability of cancer-related transcripts. Aberrant overexpression of HuR has been already reported in several types of tumors [35,36,37]. Here, we describe that the RBP UNR/*CSDE1* is associated to cell survival, invasion, resistance to apoptosis, and poor prognosis in CRC through the regulation of EMT and c-MYC expression. Firstly, we observed UNR/*CSDE1* overexpression in a series of CRC-derived cell lines and paired human colon biopsies that supported its oncogenic role. Subsequently, UNR/*CSDE1* was found to be highly expressed in colonspheres compared to their parental cell lines. This result supports the role of UNR/*CSDE1* in the regulation of cell differentiation that has been previously described in mouse embryonic stem cells [38]. Furthermore, our study highlights the invasive phenotype due the higher expression of UNR/*CSDE1* in metastatic cell lines compared to primary cell lines, and the reduced migration abilities of cell lines after UNR downregulation. These functions of UNR/*CSDE1,* which involve invasive phenotype, are supported by a previous report in melanoma derived cell lines and tumors [15]. All these results demonstrate that UNR/*CSDE1* expression is a crucial factor associated with the acquisition of molecular traits involved in invasiveness and metastasis.

It has been reported that UNR/*CSDE1* downregulation induces apoptosis in untreated and gamma-irradiated cells [39]. Then, we aimed to evaluate whether UNR downregulation could influence apoptosis mediated by camptothecin [40]. Although UNR downregulation *per se* was not enough to produce significant apoptotic stimulus in both studied CRC derived cell lines, UNR downregulation potentiated the apoptotic effect of this drug. However, further studies are required to propose UNR/*CSDE1* as a target for future drug design in cancer.

Finally, we then aimed to identify the prognostic value of UNR/*CSDE1* in CRC patients. Although a high trend toward significance was found between UNR expression and survival, it did not achieve statistical significance. This result was reasonably expected due the scarce sample size of cohort I. We then assessed a second analysis in high number of CRC patients (cohort II), and a significant prognostic relevance of high levels of UNR appeared in both progression-free and overall survival analysis. However, the role of UNR/*CSDE1* as a prognosis biomarker appears to be different depending on the type tumor [18]. From our experiments we can conclude that high expression of UNR/*CSDE1* is associated to poor prognosis in CRC.

In order to explain the malignant phenotype conferred by UNR/*CSDE1* expression in CRC, we evaluated other proteins that have been previously linked to UNR/*CSDE1*. It has been previously reported how UNR/*CSDE1* regulates direct translation of vimentin in melanoma cells [15]. Vimentin expression is involved in acquisition of mesenchymal phenotype that induces cell migration, invasion and metastasis in cancer [41]. Other EMT proteins have been evaluated to verify the role of UNR in acquisition of mesenchymal phenotype and migration abilities of tumor cells. Slug and snail are a set of transcription factors involved in EMT induced by TGF-β [42,43]. Moreover, β-catenin expression has been associated to mesenchymal phenotype in CRC [44]. In contrast, cytokeratin 20 expression is a routine test to detect epithelial neoplasms [45]. Here, we showed downregulation of mesenchymal markers snail, slug, TGF-β and β-catenin, and upregulation of epithelial marker cytokeratin 20 after UNR downregulation. Moreover, downregulation of TGF-β after UNR downregulation goes in accordance with previous findings that reported the direct bind of UNR to TGFB1 [15]. These results support the role of UNR in EMT in this type of tumor.

Subsequently, from all related targets of UNR/*CSDE1* described in the literature, we have focused in c-MYC. On the one hand, c-MYC is a proto-oncogen involved in tumor development [46,47,48]. On the other hand, not only is UNR/*CSDE1* closely related to c-MYC, since UNR stimulates IRES-dependent translation of c-myc mRNA [11,14], but c-MYC is also able to repress UNR expression, which may explain the auto-regulatory feedback between both proteins [49]. In addition, the role of c-MYC has been also related to chemoresistance and anti-apoptotic effect in different tumor types against different treatments [50,51,52,53,54], and c-MYC expression has been directly related to mesenchymal shift during carcinogenesis [55]. In this study, we observed that downregulation of UNR led to a reduced c-MYC protein levels in vitro, and we found a moderate significant positive correlation between UNR and c-MYC expression in CRC patients. Consequently, mesenchymal reversion and the increased apoptosis after UNR downregulation could be explained with the regulation of c-MYC protein expression. Additionally, poor prognosis observed in CRC patients was associated with high UNR expression, which may be justified by the link between UNR and c-MYC, since c-MYC expression has been associated which poor prognosis in other types of tumors [56,57,58].

Since UNR/*CSDE1* has an important role in tumorigenesis, and its expression levels could predict the outcome of CRC patients, UNR targeting constitutes a promising approach for drug discovery and development to be evaluated in future pre-clinical and clinical trials, both alone and in combination with other targeted therapies.

## 5. Conclusions

This study has shown that UNR/*CSDE1* is involved in the malignant phenotype of tumor cells by regulation of EMT and c-MYC expression. In this respect, UNR engrosses the currently small list of RNA binding proteins, including HuR, eIF4E, and Sam68, whose aberrant expression contribute to the cancerous phenotype.

## Figures and Tables

**Table 1:** Clinico-pathological characteristics of CRC patients from training set included in the study.

Characteristics	N (%)	Characteristics	N (%)
Median Age (range)	64 years (37–83)	Mestastasic disease	
Gender		Synchronous	23 (66%)
Female	13 (37%)	Metachronous	12 (34%)
Male	18 (52%)	Differentiation grade	
N/A	4 (11%)	Well	9 (26%)
Family history of cancer		Moderate	16 (46%)
No	25 (72%)	Poor	3 (8%)
Yes	5 (14%)	N/A	7 (20%)
N/A	5 (14%)	Inflammation	
Localization of primary tumor		No	11 (32%)
Right	6 (17%)	Low	14 (40%)
Transverse	2 (6%)	Moderate	4 (11%)
Left	2 (6%)	N/A	6 (17%)
Sigma	11 (32%)	Vascular Invasion	
Rectum	8 (23%)	No	25 (72%)
Cecum	3 (8%)	Yes	4 (11%)
N/A	3 (8%)	N/A	6 (17%)
pT		MMR	
T1	2 (6%)	Deficient	3 (9%)
T2	2 (6%)	Proficient	29 (82%)
T3	29 (82%)	N/A	3 (9%)
T4	1 (3%)	RAS status	
N/A	1 (3%)	wild-type	24 (69%)
pN		Mutated	6 (17%)
N0	18 (52%)	N/A	5 (14%)
N1	11 (31%)	UNR	
N2	5 (14%)	High	14 (40%)
N/A	1 (3%)	Low	21 (60%)

N: number of patients; pT: tumor infiltration; pN: lymph node involvement; MMR: mismatch-repair gene status; N/A: not available.

## Supplementary Materials

The following are available online at https://www.mdpi.com/2077-0383/8/4/560/s1, Figure S1. ROC curve of the analyzed training set according to UNR Hscore. (A) Table containing ROC curve estimation parameters. AUC: area under the curve. (B) ROC curve performed with UNR Hscore.

## References

[1] Siegel R.L., Miller K.D., Jemal A. (2019). Cancer statistics, 2019. CA Cancer J. Clin..

[2] Brenner H., Chen C. (2018). The colorectal cancer epidemic: Challenges and opportunities for primary, secondary and tertiary prevention. Br. J. Cancer.

[3] Fearon E.R., Vogelstein B. (1990). A genetic model for colorectal tumorigenesis. Cell.

[4] Markowitz S.D., Bertagnolli M.M. (2009). Molecular Basis of Colorectal Cancer. N. Engl. J. Med..

[5] Wurth L. (2012). Versatility of RNA-binding proteins in cancer. Comp. Funct. Genom..

[6] Van Kouwenhove M., Kedde M., Agami R. (2011). MicroRNA regulation by RNA-binding proteins and its implications for cancer. Nat. Rev. Cancer.

[7] Jeffers M., Paciucci R., Pellicer A. (1990). Characterization of unr; a gene closely linked to N-ras. Nucleic Acids Res..

[8] Boussadia O., Amiot F., Cases S., Triqueneaux G., Jacquemin-Sablon H., Dautry F. (1997). Transcription of unr (upstream of N-ras) down-modulates N-ras expression in vivo. FEBS Lett..

[9] Martín-Hernández J., Sørensen A.B., Pedersen F.S. (2001). Murine Leukemia Virus Proviral Insertions between the N-ras and unr Genes in B-Cell Lymphoma DNA Affect the Expression of N-ras Only. J. Virol..

[10] Triqueneaux G., Velten M., Franzon P., Dautry F., Jacquemin-Sablon H. (1999). RNA binding specificity of Unr, a protein with five cold shock domains. Nucleic Acids Res..

[11] Mitchell S.A., Spriggs K.A., Coldwell M.J., Jackson R.J., Willis A.E. (2003). The Apaf-1 internal ribosome entry segment attains the correct structural conformation for function via interactions with PTB and unr. Mol. Cell.

[12] Grosset C., Chen C.Y.A., Xu N., Sonenberg N., Jacquemin-Sablon H., Shyu A. (2000). Bin A mechanism for translationally coupled mRNA turnover: Interaction between the poly(A) tail and a c-fos RNA coding determinant via a protein complex. Cell.

[13] Chang T.C., Yamashita A., Chen C.Y.A., Yamashita Y., Zhu W., Durdan S., Kahvejian A., Sonenberg N., Shyu A. (2004). Bin UNR, a new partner of poly(A)-binding protein, plays a key role in translationally coupled mRNA turnover mediated by the c-fos major coding-region determinant. Genes Dev..

[14] Evans J.R., Mitchell S.A., Spriggs K.A., Ostrowski J., Bomsztyk K., Ostarek D., Willis A.E. (2003). Members of the poly (rC) binding protein family stimulate the activity of the c-myc internal ribosome entry segment in vitro and in vivo. Oncogene.

[15] Wurth L., Papasaikas P., Olmeda D., Bley N., Calvo G.T., Guerrero S., Cerezo-Wallis D., Martinez-Useros J., García-Fernández M., Hüttelmaier S. (2016). UNR/CSDE1 Drives a Post-transcriptional Program to Promote Melanoma Invasion and Metastasis. Cancer Cell.

[16] Sun X., Fang H., Li X., Rossin R., Welch M.J., Taylor J.S. (2005). MicroPET imaging of MCF-7 tumors in mice via unr mRNA-targeted peptide nucleic acids. Bioconjug. Chem..

[17] Zhang C., Zhang M., Wu Q., Peng J., Ruan Y., Gu J. (2015). Hepsin inhibits CDK11p58 IRES activity by suppressing unr expression and eIF-2α phosphorylation in prostate cancer. Cell. Signal..

[18] Martinez-Useros J., Georgiev-Hristov T., Fernández-Aceñero M.J., Borrero-Palacios A., Indacochea A., Guerrero S., Li W., Cebrián A., Del Pulgar T.G., Puime-Otin A. (2017). UNR/CDSE1 expression as prognosis biomarker in resectable pancreatic ductal adenocarcinoma patients: A proof-of-concept. PLoS ONE.

[19] Bitarte N., Bandres E., Boni V., Zarate R., Rodriguez J., Gonzalez-Huarriz M., Lopez I., Javier Sola J., Alonso M.M., Fortes P. (2011). MicroRNA-451 is involved in the self-renewal, tumorigenicity, and chemoresistance of colorectal cancer stem cells. Stem Cells.

[20] Souza V., Dong Y. Bin, Zhou H.S., Zacharias W., McMasters K.M. (2005). SW-620 cells treated with topoisomerase I inhibitor SN-38: Gene expression profiling. J. Transl. Med..

[21] Martinez-Useros J., Rodriguez-Remirez M., Borrero-Palacios A., Moreno I., Cebrian A., Gomez del Pulgar T., del Puerto-Nevado L., Vega-Bravo R., Puime-Otin A., Perez N. (2014). DEK is a potential marker for aggressive phenotype and irinotecan-based therapy response in metastatic colorectal cancer. BMC Cancer.

[22] Fuhrich D.G., Lessey B.A., Savaris R.F. (2013). Comparison of HSCORE assessment of endometrial β3 integrin subunit expression with digital HSCORE using computerized image analysis (ImageJ). Anal. Quant. Cytol. Histol..

[23] Cohen L.H., Cohen L.H. (1988). Measurement of life events. Life Events and Psychological Functioning: Theoretical and Methodological Issues.

[24] Gao J., Aksoy B.A., Dogrusoz U., Dresdner G., Gross B., Sumer S.O., Sun Y., Jacobsen A., Sinha R., Larsson E. (2013). Integrative analysis of complex cancer genomics and clinical profiles using the cBioPortal. Sci. Signal..

[25] Cerami E., Gao J., Dogrusoz U., Gross B.E., Sumer S.O., Aksoy B.A., Jacobsen A., Byrne C.J., Heuer M.L., Larsson E. (2012). The cBio Cancer Genomics Portal: An open platform for exploring multidimensional cancer genomics data. Cancer Discov..

[26] Weiswald L.B., Richon S., Validire P., Briffod M., Lai-Kuen R., Cordelières F.P., Bertrand F., Dargere D., Massonnet G., Marangoni E. (2009). Newly characterised ex vivo colospheres as a three-dimensional colon cancer cell model of tumour aggressiveness. Br. J. Cancer.

[27] Wang X., Gong W., Qing H., Geng Y., Wang X., Zhang Y., Peng L., Zhang H., Jiang B. (2010). P21-activated kinase 5 inhibits camptothecin-induced apoptosis in colorectal carcinoma cells. Tumor Biol..

[28] Matter N., Herrlich P., König H. (2002). Signal-dependent regulation of splicing via phosphorylation of Sam68. Nature.

[29] Busà R., Paronetto M.P., Farini D., Pierantozzi E., Botti F., Angelini D.F., Attisani F., Vespasiani G., Sette C. (2007). The RNA-binding protein Sam68 contributes to proliferation and survival of human prostate cancer cells. Oncogene.

[30] Rajan P., Gaughan L., Dalgliesh C., El-Sherif A., Robson C.N., Leung H.Y., Elliott D.J. (2008). Regulation of gene expression by the RNA-binding protein Sam68 in cancer. Biochem. Soc. Trans..

[31] Topisirovic I., Sonenberg N. (2011). mRNA translation and energy metabolism in cancer: The role of the MAPK and mtorc1 Pathways. Cold Spring Harb. Symp. Quant. Biol..

[32] Hsieh A.C., Ruggero D. (2010). Targeting eukaryotic translation initiation factor 4E (eIF4E) in cancer. Clin. Cancer Res..

[33] Coleman L.J., Peter M.B., Teall T.J., Brannan R.A., Hanby A.M., Honarpisheh H., Shaaban A.M., Smith L., Speirs V., Verghese E.T. (2009). Combined analysis of eIF4E and 4E-binding protein expression predicts breast cancer survival and estimates eIF4E activity. Br. J. Cancer.

[34] Dumstorf C.A., Konicek B.W., McNulty A.M., Parsons S.H., Furic L., Sonenberg N., Graff J.R. (2010). Modulation of 4E-BP1 Function as a Critical Determinant of Enzastaurin-Induced Apoptosis. Mol. Cancer Ther..

[35] Bergalet J., Fawal M., Lopez C., Desjobert C., Lamant L., Delsol G., Morello D., Espinos E. (2011). HuR-Mediated Control of C/EBP mRNA Stability and Translation in ALK-Positive Anaplastic Large Cell Lymphomas. Mol. Cancer Res..

[36] Kakuguchi W., Kitamura T., Kuroshima T., Ishikawa M., Kitagawa Y., Totsuka Y., Shindoh M., Higashino F. (2010). HuR Knockdown Changes the Oncogenic Potential of Oral Cancer Cells. Mol. Cancer Res..

[37] Nowotarski S.L., Shantz L.M. (2010). The RNA binding protein HuR stabilizes the ornithine decarboxylase (ODC) transcript in non-melanoma skin cancer. Cancer Res..

[38] Elatmani H., Dormoy-Raclet V., Dubus P., Dautry F., Chazaud C., Jacquemin-Sablon H. (2011). The RNA-binding protein Unr prevents mouse embryonic stem cells differentiation toward the primitive endoderm lineage. Stem Cells.

[39] Dormoy-Raclet V., Markovits J., Malato Y., Huet S., Lagarde P., Montaudon D., Jacquemin-Sablon A., Jacquemin-Sablon H. (2007). Unr, a cytoplasmic RNA-binding protein with cold-shock domains, is involved in control of apoptosis in ES and HuH7 cells. Oncogene.

[40] Hsiang Y.H., Liu L.F. (1988). Identification of mammalian dna topoisomerase i as an intracellular target of the anticancer drug camptothecin. Cancer Res..

[41] Liu C.-Y., Lin H.-H., Tang M.-J., Wang Y.-K. (2015). Vimentin contributes to epithelial-mesenchymal transition cancer cell mechanics by mediating cytoskeletal organization and focal adhesion maturation. Oncotarget.

[42] Peinado H., Olmeda D., Cano A. (2007). Snail, ZEB and bHLH factors in tumour progression: An alliance against the epithelial phenotype?. Nat. Rev. Cancer.

[43] Lamouille S., Xu J., Derynck R. (2014). Molecular mechanisms of epithelial-mesenchymal transition. Nat. Rev. Mol. Cell Biol..

[44] Chen K., Liu Q., Tsang L.L., Ye Q., Chan H.C., Sun Y., Jiang X. (2017). Human MSCs promotes colorectal cancer epithelial-mesenchymal transition and progression via CCL5/β-catenin/Slug pathway. Cell Death Dis..

[45] Chu P., Wu E., Weiss L.M. (2000). Cytokeratin 7 and Cytokeratin 20 expression in epithelial neoplasms: A survey of 435 cases. Mod. Pathol..

[46] Land H., Parada L.F., Weinberg R.A. (1983). Tumorigenic conversion of primary embryo fibroblasts requires at least two cooperating oncogenes. Nature.

[47] Keath E.J., Caimi P.G., Cole M.D. (1984). Fibroblast lines expressing activated c-myc oncogenes are tumorigenic in nude mice and syngeneic animals. Cell.

[48] Kelekar A., Cole M.D. (1986). Tumorigenicity of fibroblast lines expressing the adenovirus E1a, cellular p53, or normal c-myc genes. Mol. Cell. Biol..

[49] Anderson E.C., Catnaigh P.O. (2015). Regulation of the expression and activity of Unr in mammalian cells. Biochem. Soc. Trans..

[50] Gatti G., Maresca G., Natoli M., Florenzano F., Nicolin A., Felsani A., D’Agnano I. (2009). Myc prevents apoptosis and enhances endoreduplication induced by paclitaxel. PLoS ONE.

[51] Bucci B., D’Agnano I., Amendola D., Citti A., Raza G.H., Miceli R., De Paula U., Marchese R., Albini S., Felsani A. (2005). Myc down-regulation sensitizes melanoma cells to radiotherapy by inhibiting MLH1 and MSH2 mismatch repair proteins. Clin. Cancer Res..

[52] D’Agnano I., Valentini A., Fornari C., Bucci B., Starace G., Felsani A., Citro G. (2001). Myc down-regulation induces apoptosis in M14 melanoma cells by increasing p27kip1 levels. Oncogene.

[53] Greco C., D’Agnano I., Vitelli G., Vona R., Marino M., Mottolese M., Zuppi C., Capoluongo E., Ameglio F. (2006). c-MYC deregulation is involved in melphalan resistance of multiple myeloma: Role of PDGF-BB. Int. J. Immunopathol. Pharmacol..

[54] Pilling A.B., Kim J., Estrada-Bernal A., Zhou Q., Le A.T., Singleton K.R., Heasley L.E., Tan A.C., DeGregori J., Doebele R.C. (2018). ALK is a critical regulator of the MYC-signaling axis in ALK positive lung cancer. Oncotarget.

[55] Cho K. Bin, Cho M.K., Lee W.Y., Kang K.W. (2010). Overexpression of c-myc induces epithelial mesenchymal transition in mammary epithelial cells. Cancer Lett..

[56] Li Y., El-Kady A., Sun Y., Liao Dj. (2011). Cyclin D1 inhibits whereas c-Myc enhances the cytotoxicity of cisplatin in mouse pancreatic cancer cells via regulation of several members of the NF-κB and Bcl-2 families. J. Carcinog..

[57] Kim H.K., Choi I.J., Kim C.G., Kim H.S., Oshima A., Yamada Y., Arao T., Nishio K., Michalowski A., Green J.E. (2012). Three-gene predictor of clinical outcome for gastric cancer patients treated with chemotherapy. Pharmacogenom. J..

[58] Niimi S., Nakagawa K., Yokota J., Tsunokawa Y., Nishio K., Terashima Y., Shibuya M., Terada M., Saijo N. (1991). Resistance to anticancer drugs in NIH3T3 cells transfected with c-myc and/or c-H-ras genes. Br. J. Cancer.

