# Supplementary material for: UNR/CSDE1 Expression Is Critical to Maintain Invasive Phenotype of Colorectal Cancer through Regulation of c-MYC and Epithelial-to-Mesenchymal Transition
Source: J Clin Med. 2019 Apr 25;8(4):560. doi: 10.3390/jcm8040560 (PMC6517883; doi:10.3390/jcm8040560)
Supplement: Supplementary file 1 [file jcm-08-00560-s001.pdf]

A

UNR Hscore	Sensitivity	1 - Specificity	AUC
-1,00	1,000	1,000	0,00
5,00	,800	,500	0,40
12,50	,667	,450	0,37
17,50	,600	,450	0,33
25,00	,600	,350	0,39
35,00	,600	,250	0,45
45,00	,533	,200	0,43
55,00	,467	,100	0,42
65,00	,400	,100	0,36
75,00	,267	,100	0,24
85,00	,200	,100	0,18
115,00	,200	,050	0,19
150,00	,200	0,000	0,20
215,00	,133	0,000	0,13
277,50	,067	0,000	0,07
286,00	0,000	0,000	0,00

B

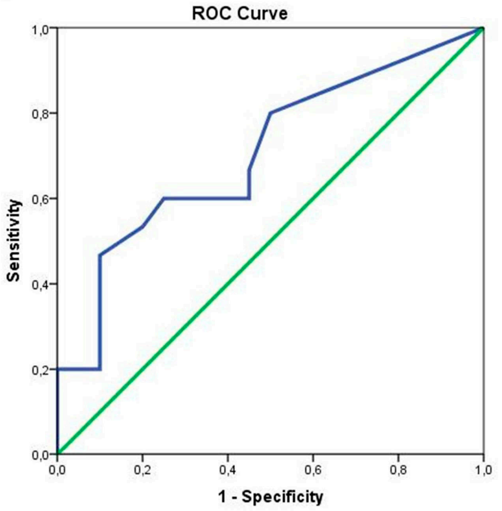

Figure 1. ROC curve of the analyzed training set according to UNR Hscore. (A) Table containing ROC curve estimation parameters. AUC: area under the curve. (B) ROC curve performed with UNR~Hscore.
